# 

**Published:** 1924-02

**Authors:** 


### HOSPITAL and HEALTH

REVIEW

Established 1886 No. 1871. Vol. LXXIII

New Series. Vol. III. No. 29 February, 1924. Price Sixpence

CONTENTS.

| | PAGE |
|---|---|
| Spiritual Healing .. .. .. .. .. .. | 35 |
| "Satellite Towns" .. .. .. .. .. .. | 36 |
| Notes and Comments :— The New Minister of Health—Hospitals and Poor Law Infirmaries—The Publicity of Inquests—The Weather and Sickness—The "London's" Latest Romance—Humane Slaughter—Legacies to Hospitals—Fighting Tuberculosis or the Tuberculous ?—White Lead—Is Appendicitis Catching ?—Hospital Ballots Old and New—Blood Transfusion—The Open-Air Nursery School—Overheated Rooms—Insulin and Experiments on Animals—Paper Containers and Health .. .. | 37 |
| Hospital Men of Mark : Colonel D. J. Mackintosh and Mr. J. Matheson Johnston .. .. | 41 |
| Some Medical Benefit Changes .. .. .. | 43 |
| Health for the Middle-aged Artisan .. .. | 44 |
| Hospital Progress and Finance .. .. | 45 |
| The Public Health : Interviews with Local Authorities : Brighton .. .. .. .. | 47 |
| The Life and Doctrine of Sir Edwin Chadwick. By Sir William Collins, K.C.V.O. .. .. .. | 50 |
| Voluntary Hospitals and Poor Law Infirmaries. By Mr. E. W. Morris, C.B.E. .. .. .. | 52 |
| Sir Napier Burnett : An Appreciation. By S. Walton | 54 |
| Reviews of Books .. .. .. .. .. .. | 55 |
| Correspondence .. .. .. .. .. .. | 58 |
| Organising a Health Week : A Prize of £100 .. | 60 |
| Echoes of the Month .. .. .. .. .. | 61 |
| Hospital and Health News .. .. .. .. | 62 |
| Answers to Correspondents .. .. .. .. | 64 |
| Recent Appointments .. .. .. .. .. | 64 |

